# The Effect of (-)-Epigallocatechin 3-*O* - Gallate *In Vitro* and *In Vivo* in *Leishmania braziliensis:* Involvement of Reactive Oxygen Species as a Mechanism of Action

**DOI:** 10.1371/journal.pntd.0003093

**Published:** 2014-08-21

**Authors:** Job D. F. Inacio, Luiza Gervazoni, Marilene M. Canto-Cavalheiro, Elmo E. Almeida-Amaral

**Affiliations:** Laboratório de Bioquímica de Tripanosomatideos, Instituto Oswaldo Cruz (IOC), Fundação Oswaldo Cruz (FIOCRUZ), Rio de Janeiro, Rio de Janeiro, Brazil; University of Alabama in Huntsville, United States of America

## Abstract

**Background:**

Leishmaniasis is a parasitic disease associated with extensive mortality and morbidity. The treatment for leishmaniasis is currently based on pentavalent antimonials and amphotericin B; however, these drugs result in numerous adverse side effects. Natural compounds have been used as novel treatments for parasitic diseases. In this paper, we evaluated the effect of (-)-epigallocatechin 3-*O*-gallate (EGCG) on *Leishmania braziliensis in vitro* and *in vivo* and described the mechanism of EGCG action against *L. braziliensis* promastigotes and intracellular amastigotes.

**Methodology/Principal Finding:**

*In vitro* activity and reactive oxygen species (ROS) measurements were determined during the promastigote and intracellular amastigote life stages. The effect of EGCG on mitochondrial membrane potential (ΔΨ_m_) was assayed using JC-1, and intracellular ATP concentrations were measured using a luciferin-luciferase system. The *in vivo* experiments were performed in infected BALB/c mice orally treated with EGCG. EGCG reduced promastigote viability and the infection index in a time- and dose-dependent manner, with IC_50_ values of 278.8 µM and 3.4 µM, respectively, at 72 h and a selectivity index of 149.5. In addition, EGCG induced ROS production in the promastigote and intracellular amastigote, and the effects were reversed by polyethylene glycol (PEG)-catalase. Additionally, EGCG reduced ΔΨ_m_, thereby decreasing intracellular ATP concentrations in promastigotes. Furthermore, EGCG treatment was also effective *in vivo*, demonstrating oral bioavailability and reduced parasitic loads without altering serological toxicity markers.

**Conclusions/Significance:**

In conclusion, our study demonstrates the leishmanicidal effects of EGCG against the two forms of *L. braziliensis*, the promastigote and amastigote. In addition, EGCG promotes ROS production as a part of its mechanism of action, resulting in decreased ΔΨ_m_ and reduced intracellular ATP concentrations. These actions ultimately culminate in parasite death. Furthermore, our data suggest that EGCG is orally effective in the treatment of *L. braziliensis*-infected BALB/c mice without altering serological toxicity markers.

## Author Summary

Leishmaniasis is a parasitic disease that is endemic in 88 countries, primarily located in tropical and subtropical regions, that affects more than 12 million people worldwide. Leishmaniasis treatments are currently based on pentavalent antimonials and amphotericin B; however, these drugs result in numerous adverse side effects and variable efficacy. In addition, the drugs are expensive, and parasite resistance to these drugs has been observed. The lack of affordable therapy necessitates the development of novel antileishmanial therapies. We investigated the antileishmanial activity of EGCG *in vitro* and *in vivo* and described the mechanism of EGCG action against *Leishmania braziliensis* promastigotes and intracellular amastigotes. EGCG reduced promastigote viability and the infection index in a time- and dose-dependent manner with a selectivity index of 149.5. This effect was reversed by polyethylene glycol (PEG)-catalase, suggesting that ROS production is a mechanism of action in promastigotes and intracellular amastigotes. Additionally, EGCG reduced ΔΨ_m_ and intracellular ATP concentrations in promastigotes. Furthermore, EGCG treatment was also effective *in vivo*, demonstrating oral bioavailability and reduced lesion sizes and parasitic load (92% of reduction) without altering serological toxicity markers. Additional studies should be conducted to determine the ideal dose and therapeutic regimen.

## Introduction

Leishmaniasis is a parasitic disease that is caused by protozoa of the genus *Leishmania* and is associated with extensive mortality and morbidity. This disease is endemic in 98 countries, mainly in tropical and subtropical regions, and affects more than 12 million people worldwide. Leishmaniasis has an annual incidence of approximately 1.3 million cases and a prevalence of approximately 350 million people living in endemic areas. The disease severity caused by various *Leishmania* species varies widely, ranging from cutaneous and/or mucosal to visceral infection [1], [2].

*Leishmania braziliensis* is the most common *Leishmania* species in the Americas and is the etiological agent of cutaneous and mucocutaneous leishmaniasis [3]. Currently, Leishmaniasis treatment is based on pentavalent antimonials and amphotericin B; however, these drugs are expensive, result in numerous adverse side effects, and exhibit variable efficacy [4]–[7].

Numerous natural compound screens have successfully identified novel treatments for parasitic diseases [8], [9]. Extracts obtained from plants and pure compounds, such as certain types of flavonoids, have been reported to possess significant antiprotozoal activity with no side effects [10]–[13]. For example, (-)-epigallocatechin 3-*O*-gallate (EGCG) is the most abundant polyphenolic flavonoid constituent of green tea and has been reported to possess anti-infective effects against viruses, bacteria and various fungi [14], anticancer properties [15], [16], proapoptotic activity [17] and antiproliferative effects on *Trypanosoma cruzi* [18] and *Leishmania amazonensis* [19]. Although the precise molecular mechanism of action for EGCG is not yet known, EGCG has been shown to induce mitochondrial damage [20] and the production of superoxide anions, hydrogen peroxide, and other reactive oxygen species (ROS) [21]–[24].

In this study, we investigated the antileishmanial activity of EGCG *in vitro* and *in vivo* and described its mechanism of action against *Leishmania braziliensis* promastigotes and intracellular amastigotes. EGCG inhibited promastigote and intracellular amastigote proliferation in a dose-dependent manner. Additionally, EGCG was non-cytotoxic to murine macrophages at the concentration that induced potent leishmanicidal activity. This leishmanicidal activity was ROS-dependent, thus promoting mitochondrial dysfunction and reduced intracellular ATP concentrations. EGCG treatment was also effective in a murine model of *Leishmania braziliensis* infection, demonstrating oral bioavailability and decreased parasitic load without altering serological toxicology markers, such as aminotransferases and creatinine.

## Materials and Methods

### Reagents

Schneider's *Drosophila* medium, (-)-epigallocatechin 3-*O*-gallate (EGCG), fetal calf serum, penicillin, streptomycin, horseradish peroxidase, and RPMI 1640 medium were obtained from Sigma-Aldrich (St. Louis, MO, USA). H_2_DCFDA (2′,7′-dichlorodihydrofluorescein diacetate), Amplex Red, and Alamar-Blue were obtained from Invitrogen Molecular Probes (Leiden, The Netherlands). All other reagents were purchased from Merck (São Paulo, Brazil). The deionized, distilled water was obtained using a Milli-Q system of resins (Millipore Corp., Bedford, MA, USA) and used in the preparation of all solutions. Endotoxin-free, sterile disposables were used in all experiments. EGCG was prepared in phosphate-buffered saline (PBS, pH 7.2)

### Parasites

*L. braziliensis* promastigotes (MCAN/BR/97/P142 strain) were grown at 26°C (pH 7.2) in Schneider's *Drosophila* medium supplemented with 100 U/ml penicillin, 100 µg/ml streptomycin, 20% (v/v) heat-inactivated fetal calf serum and 2% sterile human urine. The parasite number was determined by direct counting using a Neubauer chamber.

### Cell proliferation

*L. braziliensis* promastigotes (MCAN/BR/97/P142 strain) were seeded into fresh medium containing Schneider's *Drosophila* medium (1.0 ml final volume) supplemented with 100 U/ml penicillin, 100 µg/ml streptomycin, 20% (v/v) heat-inactivated fetal calf serum and 2% sterile human urine either in the absence (10 µl PBS) or presence of various EGCG concentrations (10 µl; 62.5–500 µM). The cells were maintained for 72 h at 26°C. The cell density was estimated using a Neubauer chamber. The growth curve was initiated with 1.0×10^6^ cells/ml. The 50% inhibitory concentration (IC_50_) was determined by logarithmic regression analysis using GraphPad Prism 5 (GraphPad Software, La Jolla, CA, USA).

### Hydrogen peroxide production

Hydrogen peroxide production was measured using Amplex red and horseradish peroxidase (HRP) [25]. Promastigotes were treated for 72 h in the absence or presence of EGCG (62.5–500 µM). Cells were harvested and resuspended in HBSS. The cell number was obtained by counting using a Neubauer chamber. Promastigotes (2×10^7^ cells/mL) were incubated with HBSS containing 10 µM Amplex red reagent and 10 U/ml HRP. Digitonin (64 µM) was added to permeabilize the parasites. Fluorescence was monitored at excitation and emission wavelengths of 560 and 590 nm, respectively, in a spectrofluorimeter. Calibration was performed using known quantities of H_2_O_2_. Data are expressed as the fold increase in hydrogen peroxide production relative to the control.

### Determination of mitochondrial membrane potential (ΔΨ_m_)

The cationic probe JC-1 was used to determine the mitochondrial membrane potential (ΔΨ_m_) as described [13]. Promastigotes (1×10^6^ cells/ml) were cultured for 72 h in the absence or presence of 62.5–500 µM EGCG. Cells were harvested and re-suspended in Hank's Balanced Salt Solution (HBSS). The cell number was obtained via counting in a Neubauer chamber. Promastigotes (1×10^7^ cells/ml) were incubated with JC-1 (10 µg/ml) for 10 minutes at 37°C. After washing twice with HBSS, fluorescence was measured spectrofluorometrically at 530 nm and 590 nm using an excitation wavelength of 480 nm. The ratio of values obtained at 590 nm and 530 nm was plotted as the relative ΔΨ_m_. The mitochondrial uncoupling agent carbonyl cyanide *p*-trifluoromethoxyphenylhydrazone (FCCP; 20 µM) was used as a positive control.

### Intracellular ATP concentration measurement

Intracellular ATP concentrations were measured in treated and untreated cells using a CellTiter-Glo luminescent assay (Promega), where the signal is proportional to the ATP concentration. Briefly, promastigotes were treated for 72 h in the absence or presence of EGCG (62.5–500 µM). The cultures were washed thrice, and the parasite concentration was adjusted to 1×10^7^ cells in 200 µl of PBS. A 50-µl aliquot of each sample was transferred to a 96-well plate and mixed with the same volume of CellTiter-Glo. The plates were incubated in the dark for 10 min, and the bioluminescence was measured using a GloMax-Multi Microplate Multimode Reader (Promega). ATP concentrations were calculated from the ATP standard curve.

### *Leishmania*-macrophage interaction assay

*L. braziliensis* promastigotes were washed with phosphate buffered saline (PBS). The number of promastigotes was determined by counting with a Neubauer chamber. The promastigotes were added to the peritoneal macrophages at a parasite ratio of 3∶1. The macrophages were collected from Swiss mice (6–8 weeks old) and plated in RPMI at a concentration of 2×10^6^ cells/ml (0.4 ml/well) in Lab-Tek eight-chamber slides. This mixture was then incubated for 3 h at 37°C in a 5% CO_2_ atmosphere. The free parasites were removed by successive washes with PBS. *Leishmania*-infected macrophages were then incubated in either the absence or presence of EGCG (3 µM, 6 µM and 12 µM) for 24 and 72 h. The percentage of infected macrophages was determined by light microscopy and random counts of a minimum of 300 cells on each coverslip in duplicate. The results were expressed as an infection index (% of infected macrophages×number of amastigotes/total number of macrophages). The IC_50_ was determined by logarithmic regression analysis using GraphPad Prism 5. Pentamidine (12 µM) was used as a reference drug.

### Viability assay

Peritoneal macrophages (2×10^6^ cell/ml) collected from Swiss mice (6–8 weeks old) were allowed to adhere in black 96-well tissue culture plates for 1 h at 37°C in a 5% CO_2_ atmosphere. The non-adherent cells were removed by washes with RPMI 1640 medium, and the wells containing adherent macrophages were refilled with RPMI 1640 medium supplemented with 10% fetal bovine serum. Increasing EGCG concentrations (3 to 3000 µM) were added to the cell culture for 24 and 72 h. The medium was then discharged, and the macrophages were washed with RPMI 1640 medium. Alamar-Blue (10% v/v) was added for 12 h at 37°C in a 5% CO_2_ atmosphere. The absorbance was measured at 570 nm with a spectrophotometer. IC_50_ values were determined by logarithmic regression analysis using GraphPad Prism 5. The selectivity index was determined using the following equation: macrophage IC_50_/intracellular amastigote IC_50_, as described by Weniger et al. [26]. Peritoneal macrophages were lysed with 0.1% Triton X-100 and used as positive controls.

### Measurement of ROS levels in *Leishmania*-infected macrophages

Intracellular ROS levels were measured in promastigotes, non-*Leishmania*-infected macrophages and *Leishmania*-infected macrophages treated and untreated with EGCG. *L. braziliensis* promastigotes were washed with PBS and counted using a Neubauer chamber. The promastigotes were added to peritoneal macrophages collected from Swiss mice (6–8 weeks old) at a parasite ratio of 3∶1, and the cells were plated in black 96-well tissue culture plates at a cellular density of 2×10^6^ macrophages/ml. This mixture was then incubated for 3 h at 37°C in a 5% CO_2_ atmosphere. The free parasites were removed by successive washes with PBS. For the non-*Leishmania*-infected macrophages, peritoneal macrophages were collected from Swiss mice (6–8 weeks old) and plated in black 96-well tissue culture plates at a cellular density of 2×10^6^ macrophages/ml. The cells were incubated for 3 h at 37°C in a 5% CO_2_ atmosphere. Non-*Leishmania*-infected macrophages and *Leishmania*-infected macrophages were incubated in the absence or presence of EGCG (12 µM) for 24 h followed by H_2_DCFDA (20 µM) for 30 minutes at 37°C. The fluorescence was measured spectrofluorometrically at 530 nm using an excitation wavelength of 507 nm. For all measurements, the basal fluorescence was subtracted. The positive control was obtained by the addition of 20 units/ml glucose oxidase+60 mM glucose for 20 minutes.

### *In vivo* infection in the murine model

BALB/c mice (5/group) were maintained under specific pathogen-free conditions and then inoculated with stationary-phase *L. braziliensis* promastigote (2×10^6^ cells in 10 µl of PBS) intradermally in the right ear using a 27.5-gauge needle. The method of treatment was similar to previously described methods [27], [28] and initiated 21 days following infection. EGCG (100 mg/kg/day) was diluted in PBS and administered orally once daily seven times a week until the end of the experiment (day 32) when the animals were euthanized. The control group was treated orally with sterile PBS. The positive control was treated with intraperitoneal injections of meglumine antimoniate (30 mg/kg/day) once daily seven times a week until the end of the experiment (day 32). The lesion sizes were measured twice a week using a dial caliper.

### Parasite load quantification

The parasite load was determined 32 days post-infection using a quantitative limiting dilution assay, as previously described [29]. The infected ears were excised, weighed and minced in Schneider's medium with 20% fetal calf serum. The resulting cell suspension was serially diluted. The number of viable parasites in each ear was estimated from the highest dilution that promoted promastigote growth after 7 days of incubation at 26°C.

### Toxicology

The serum levels of aspartate aminotransferase (AST), alanine aminotransferase (ALT) and creatinine in the infected BALB/c mice treated orally and intraperitoneally as described above were measured using laboratory colorimetric kits (Doles, Goiânia, Brazil).

### Ethics statement

This study was performed in strict accordance with the recommendations of the Guide for the Care and Use of Laboratory Animals of the Fundação Oswaldo Cruz. The protocol was approved by the Committee on the Ethics of Animal Experiments of the Fundação Oswaldo Cruz (License Number: LW-7/10).

### Statistical analysis

All experiments were performed thrice. The data were analyzed statistically using Student's t-test and a one-way or two-way analysis of variance (ANOVA) followed by Bonferroni's post-test using GraphPad Prism 5 (GraphPad Software, La Jolla, CA, USA). The results were considered significant when *p*≤0.05. The data are expressed as the mean ± standard error.

## Results

### The effect of (-)-epigallocatechin 3-*O*-gallate (EGCG) on *Leishmania braziliensis* promastigotes is dose-dependent

**Figure 1:**
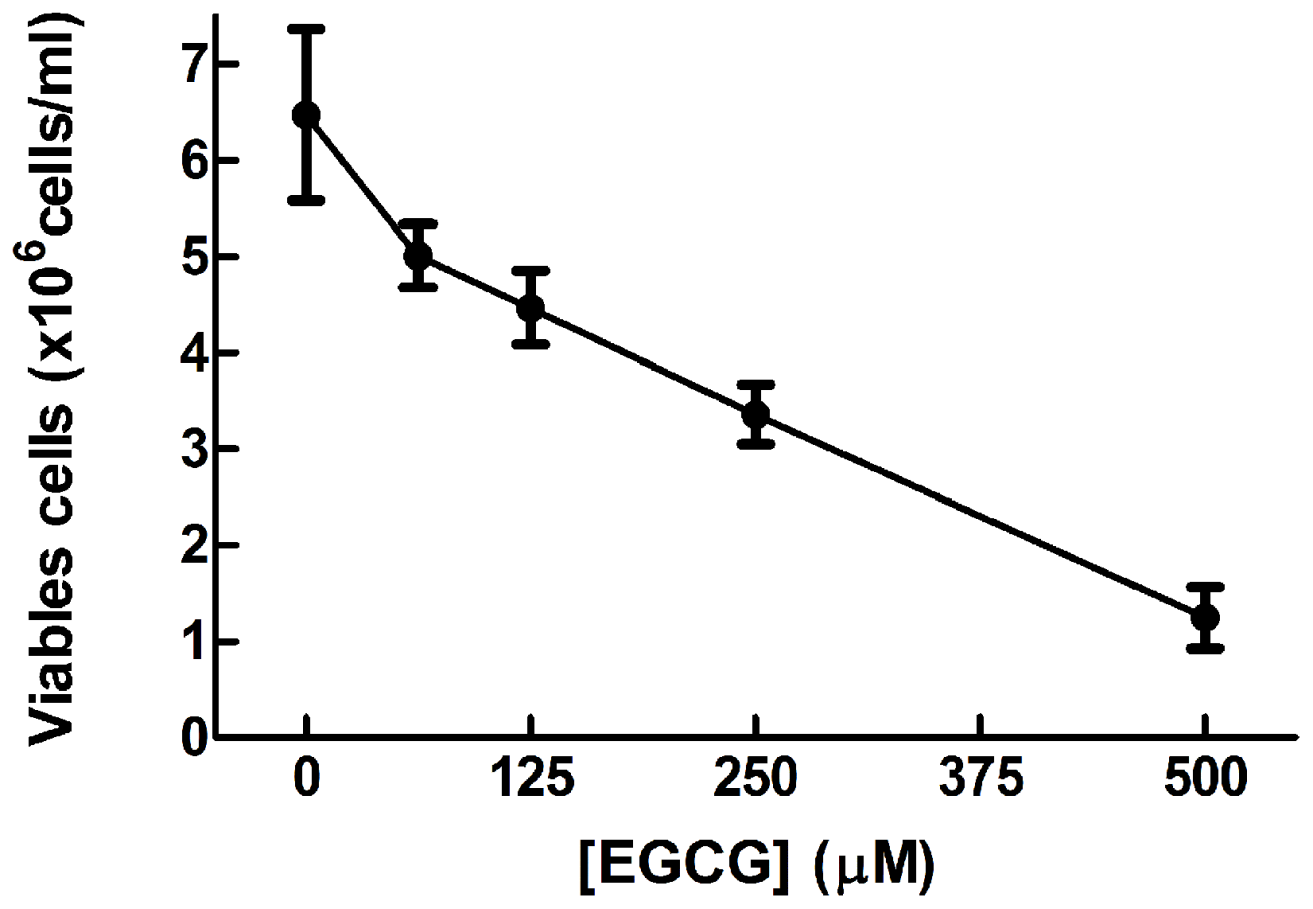
The effect of EGCG on *L. braziliensis* promastigotes. *L. braziliensis* was cultivated in Schneider's *Drosophila* medium at 26°C for 72 h in the absence or presence of EGCG (62.5–500 µM). The number of parasites was determined by direct counting using a Neubauer chamber. In the control (absence of EGCG), the same volume of PBS (solvent of EGCG) was added to the growth medium. The values are presented as the mean ± standard error of three different experiments.

Initially, the effect of EGCG on *L. braziliensis* promastigotes was investigated. We incubated the parasites with varying EGCG concentrations (62.5–500 µM) for 72 h. EGCG decreased *L. braziliensis* promastigote viability in a dose-dependent manner (*p*<0.05) (Figure 1). The inhibitory effect was 80.7% with 0.500 mM EGCG, and the IC_50_ was 278 µM.

### EGCG promote generation of hydrogen peroxide in *L. braziliensis* promastigotes

**Figure 2:**
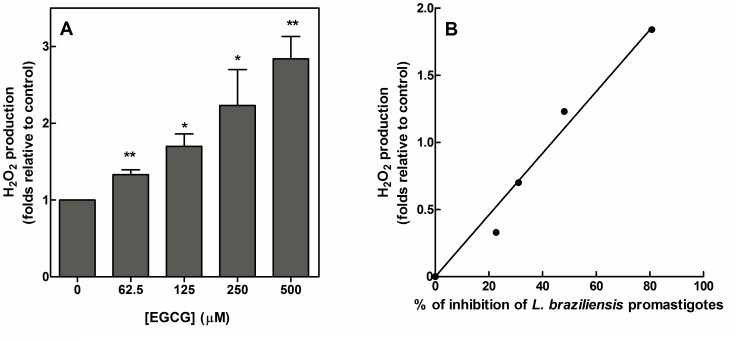
EGCG induces H_2_O_2_ formation. *Leishmania braziliensis* was cultivated in Schneider's *Drosophila* medium at 26°C as for 72 h in the absence or presence of EGCG (62.5–500 µM). H_2_O_2_ was measured using Amplex red as described in the Materials and Methods (Panel A). The data are expressed as the fold increase in H_2_O_2_ production relative to the control. The values presented are the mean ± standard error of three different experiments. * indicates a significant difference relative to the control group (*p*<0.05); ** indicates a significant difference relative to the control group (*p*<0.01). Panel B: Correlation between the H_2_O_2_ production and inhibition of *L. braziliensis* viability by EGCG (R^2^ = 0.975).

EGCG induces hydrogen peroxide (H_2_O_2_) production in various biological contexts [30]. Therefore, we investigated whether EGCG-mediated H_2_O_2_ generation in *L. braziliensis* promastigotes is a possible mechanism of cell death. EGCG treatment for 72 h increased H_2_O_2_ generation in *L. braziliensis* in a dose-dependent manner (*p*<0.01) (Figure 2A). The ROS levels were 2.9-fold higher in *L. braziliensis* treated with 500 µM EGCG compared with the control. A linear correlation (R^2^ = 0.975) between the percent inhibition of the infection index and EGCG-mediated H_2_O_2_ production was observed (Figure 2B).

**Figure 3:**
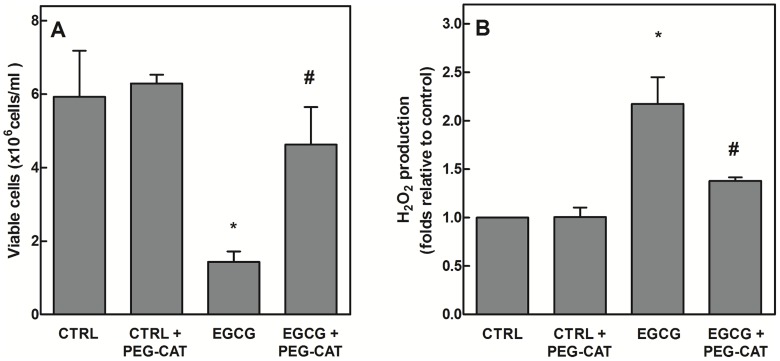
The effect of PEG-catalase on EGCG-induced cell death (A) and H_2_O_2_ formation (B). *L. braziliensis* was cultivated in Schneider's *Drosophila* medium at 26°C for 72 h with PEG-catalase in the absence or presence of EGCG as described in the Materials and Methods. Final concentrations of 500 U/ml PEG-catalase and 500 µM EGCG were added to the culture. The values presented are the mean ± standard error of three different experiments. In the control (absence of EGCG), the same volume of vehicle (PBS) was added to the growth medium. H_2_O_2_ was measured with Amplex red as described in the Materials and Methods. The data are expressed as the fold increase in H_2_O_2_ production relative to the control. The values are presented as the mean ± standard error of three different experiments. CTRL, control; PEGCAT, 500 U/ml Peg-catalase. * indicates a significant difference relative to the control group (*p*<0.05); # indicates a significant difference relative to the EGCG-treated group (*p*<0.05).

To confirm that the inhibitory effects of EGCG are mediated by H_2_O_2_ production, we pre-incubated *L. braziliensis* promastigotes with polyethylene glycol (PEG)-catalase (500 U/ml), which catalyzes hydrogen peroxide to water and oxygen. (PEG)-catalase protected *L. braziliensis* from EGCG-mediated effects (Figure 3A) and reduced H_2_O_2_ levels in EGCG-treated cells (Figure 3B), suggesting that H_2_O_2_ production is a possible mechanism for the induction of *L. braziliensis* promastigote death.

### EGCG induces mitochondrial membrane potential (ΔΨ_m_) depolarization in *Leishmania braziliensis*

**Figure 4:**
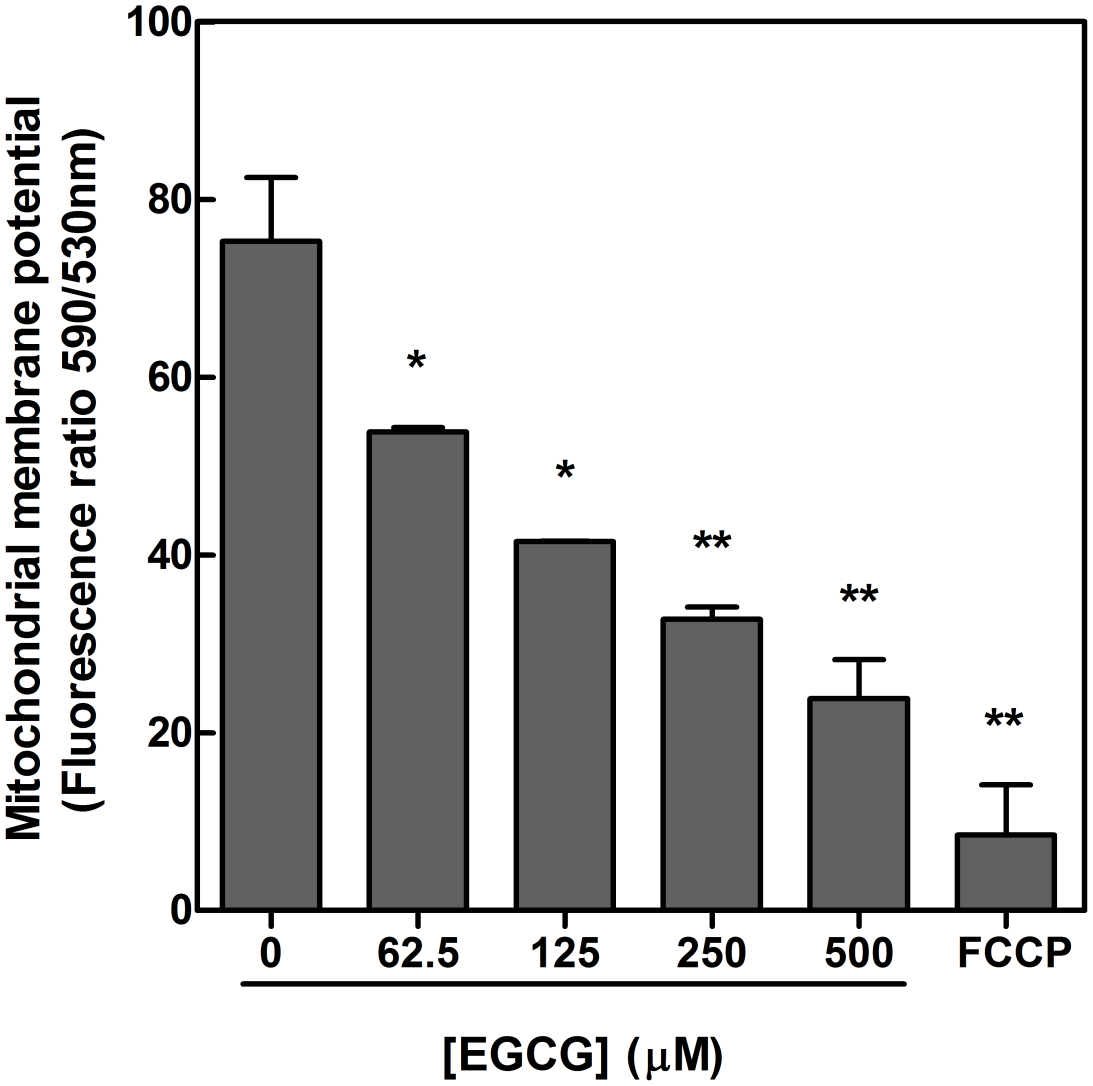
The effect of EGCG on mitochondrial membrane potential in *Leishmania braziliensis*. *Leishmania braziliensis* was cultivated in Schneider's *Drosophila* medium at 26°C for 72 h in the absence or presence of 62.5–500 µM EGCG. Promastigotes were labeled with the potentiometric probe JC-1 (10 µg/ml). The positive control was treated with FCCP (20 µM) for 20 minutes. In the control (absence of EGCG), the same volume of vehicle (PBS) was added to the growth medium. Dose-dependent alterations in relative ΔΨ_m_ values are expressed as the ratio of the fluorescence measurements at 590 nm (for J-aggregate) versus 530 nm (for J-monomer). The data are expressed as the means ± standard errors of three different experiments. * indicates a significant difference relative to the control group (*p*<0.05); ** indicates a significant difference relative to the control group (*p*<0.01).

The parasite mitochondrial function was evaluated using JC-1, a cationic mitochondrial vital dye. This dye is lipophilic and concentrates in mitochondria in proportion to the membrane potential; increased dye accumulation is observed in mitochondria with greater ΔΨ_m_. The spectrofluorometric data presented in Figure 4 indicate a marked dose-dependent decrease in the relative fluorescence intensity (ΔΨ_m_ values) (*p*<0.001). These results indicate membrane potential depolarization in cells upon treatment with 62.5 to 500 µM of EGCG, and ΔΨ_m_ was reduced by 68.4% upon treatment with 500 µM EGCG. Similarly, decreased relative fluorescence intensity values were also observed following treatment with 20 µM FCCP (88.7% reduction).

### EGCG impairs ATP production in *L. braziliensis* promastigotes

**Figure 5:**
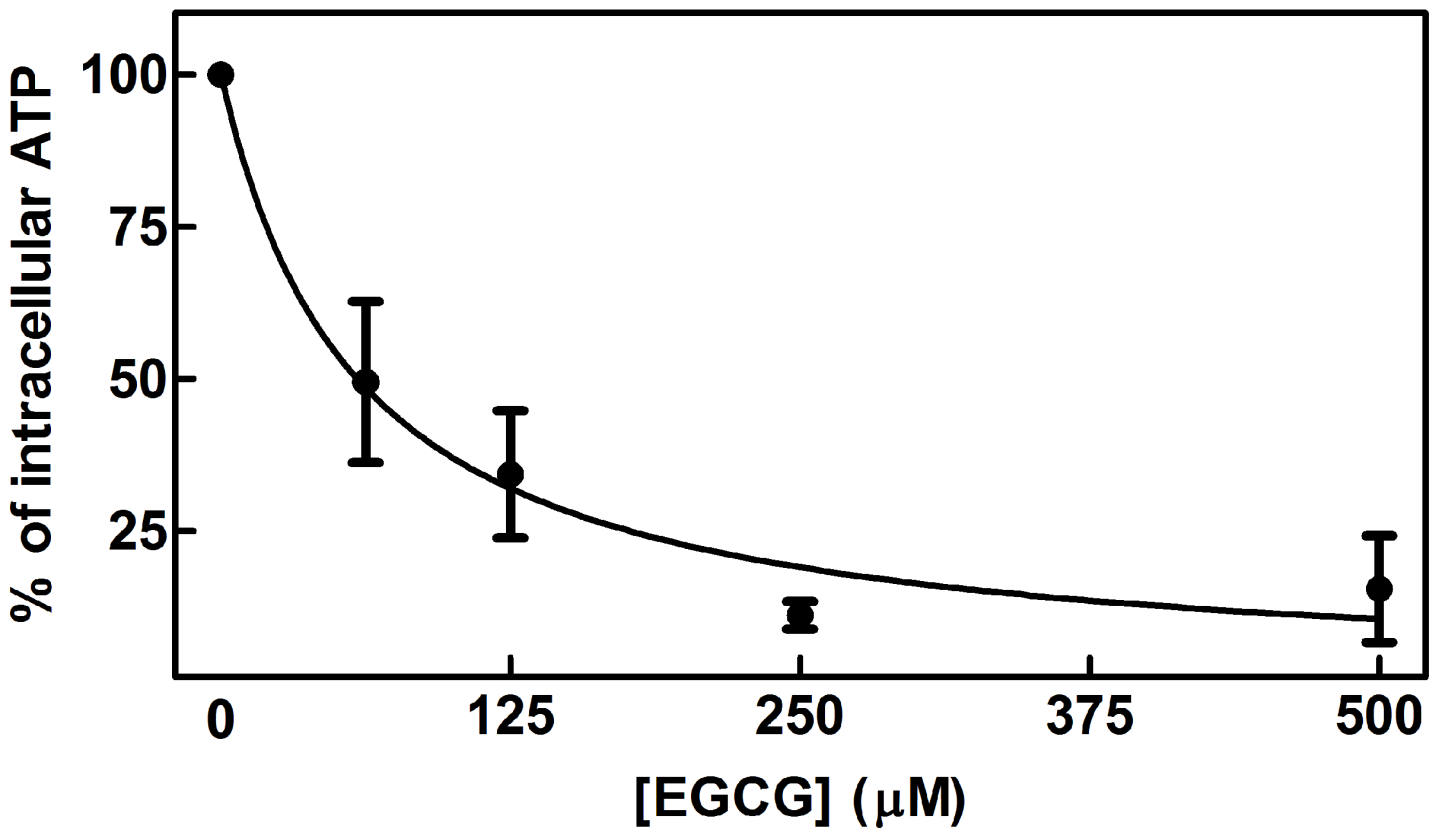
Reduced intracellular ATP concentrations in EGCG-treated *L. braziliensis* promastigotes. Promastigotes were incubated with EGCG for 72 h. Intracellular ATP concentrations were measured using a bioluminescence assay as described in the Materials and Methods. The results are expressed as a percentage of the control. The intracellular ATP concentration of the control (184.5 nmol×10^−7^ cells) was set as 100%. The values presented are the mean ± standard error of three different experiments.

Given the effect on ΔΨ_m_, we evaluated intracellular ATP concentrations in EGCG-treated parasites. EGCG reduced intracellular ATP levels in *L. braziliensis* promastigotes in a dose-dependent manner (*p*<0.001). The intracellular ATP concentration was reduced by 84.6% in parasites treated with 500 µM EGCG for 72 h (Figure 5).

### Dose-dependent effect of EGCG on *Leishmania-*infected macrophages

**Figure 6:**
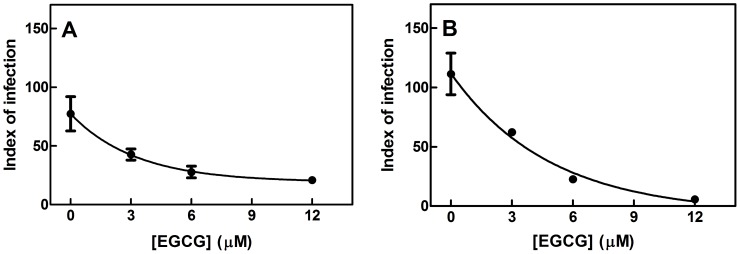
Intracellular amastigote susceptibility to EGCG. Macrophages were infected with *L. braziliensis* promastigotes for 3 h at 37°C and incubated in the absence or presence of EGCG (3 µM, 6 µM, or 12 µM) for 24 h (panel A) and 72 h (panel B). The EGCG concentrations displayed no toxic effects on the mammalian cells. The infection index was determined by light microscopy and counting at least 300 macrophages in each duplicated coverslip. In the control samples (absence of EGCG), a similar volume of vehicle (PBS) was added to the cells. The values presented are the mean ± standard error of five different experiments. Pentamidine (12 µM) was used as a reference drug and reduced the infection index by 66.9% and 94.6% after 24 and 72 h, respectively.

To determine the effects of EGCG on the interaction of *L. braziliensis* with macrophage cells after parasite invasion, untreated promastigotes were allowed to interact with macrophages for 3 h. Then, the *Leishmania*-infected macrophages were incubated in the absence or presence of EGCG (3 µM, 6 µM, or 12 µM) for 24 (Figure 6A) and 72 h (Figure 6B). EGCG reduced the infection index in a time- (*p*<0.01) and dose-dependent manner (*p*<0.001) with IC_50_ values of 3.7 and 3.4 µM, respectively. This inhibitory effect was equal to 73.0% and 94.9% with 12 µM after 24 and 72 h, respectively. The IC_50_ of EGCG against macrophages was 384.4 µM (data not shown) and 436.3 µM [19], demonstrating a selectivity index of 103.3 and 149.5 at 24 and 72 h, respectively.

### ROS production contributes to EGCG-induced death in *Leishmania*-infected macrophages

**Figure 7:**
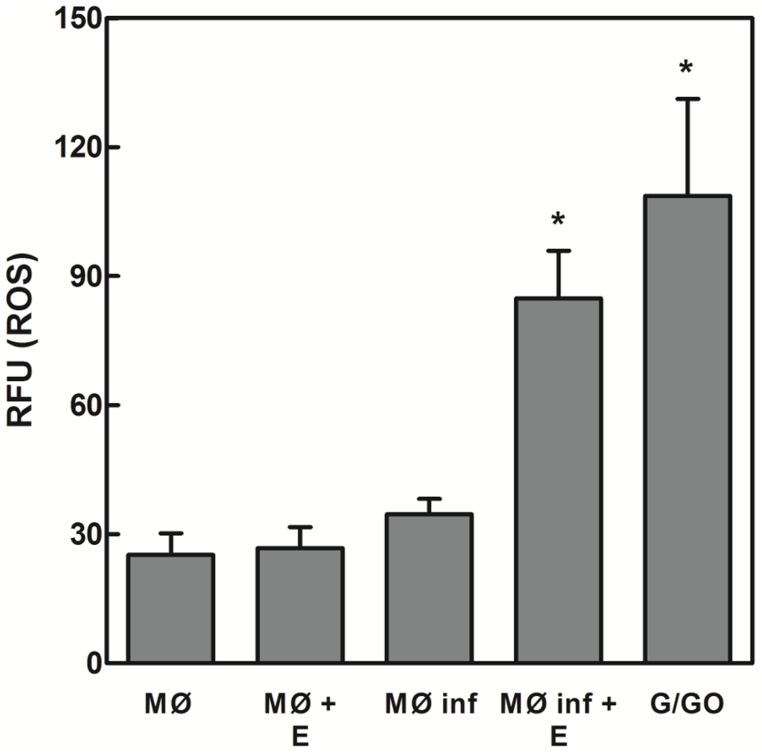
EGCG-induced ROS formation in *Leishmania*-infected macrophages. Non-*Leishmania*-infected macrophages and *Leishmania*-infected macrophages were incubated in the absence or presence of EGCG (12 µM) for 24 h. ROS were measured using the fluorescent dye H_2_DCFDA as described in the Materials and Methods. The data are expressed as fluorescence intensity units (FIU). The values presented are the mean ± standard error of three different experiments. The positive control was treated with 20 units/ml glucose oxidase and 60 mM glucose for 30 minutes. * indicates a significant difference relative to *Leishmania*-infected macrophages (*p*<0.05). MØ, non-*Leishmania*-infected macrophages; MØ+EGCG, non-*Leishmania*-infected macrophages treated with EGCG 12 µM; MØ inf, *Leishmania*-infected macrophages; MØ inf+EGCG, *Leishmania*-infected macrophages treated with EGCG 12 µM; G/GO, glucose+glucose oxidase.

EGCG possesses prooxidative properties [22]–[24]. To investigate whether the leishmanicidal effect of EGCG is due to intracellular amastigote ROS production, we measured ROS levels using the cell-permeable dye H_2_DCFDA [31]–[34]. EGCG induces ROS production in *Leishmania*-infected macrophages, not non-infected macrophages. The ROS levels were increased 2.5-fold (*p*<0.05) in EGCG-treated (12 µM) *Leishmania*-infected macrophages compared with *Leishmania*-infected macrophages throughout the experiment (Figure 7). Given that glucose oxidase catalyzes the oxidation of D-glucose and generates H_2_O_2_, this enzyme was employed as a positive control. The addition of glucose/glucose oxidase resulted in increased ROS levels compared with the control (3.1-fold, compared with ROS levels in *Leishmania*-infected macrophages).

**Figure 8:**
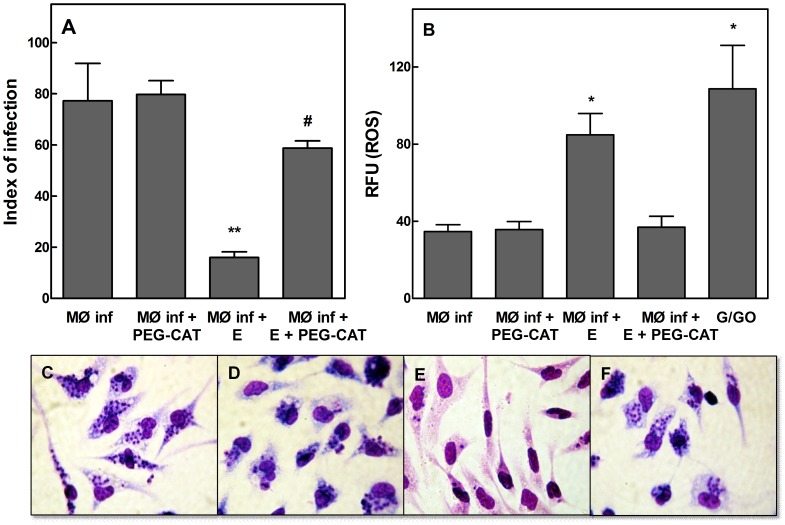
EGCG-induced leishmanicidal activity is reversed by PEG-catalase. Panel A: Macrophages were infected with *L. braziliensis* promastigotes for 3 h at 37°C as described in the Materials and Methods. The macrophages were incubated in the absence or presence of EGCG (12 µM) or PEG-catalase (500 U/ml) for 24 h. The infection index was determined using light microscopy and counting at least 300 macrophages in each duplicated coverslip. The values presented are the mean ± standard error of three different experiments. Panel B: ROS was measured using the fluorescent dye H_2_DCFDA as described in the Materials and Methods. *Leishmania*-infected macrophages were incubated in the absence or presence of EGCG (12 µM) or PEG-catalase (500 U/ml) for 24 h. The data are expressed as fluorescence intensity units (FIU). The values presented are the mean ± standard error of three different experiments. The positive control was treated with 20 units/ml glucose oxidase and 60 mM glucose for 30 minutes. MØ inf, *Leishmania*-infected macrophages; MØ inf+PEG-CAT, *Leishmania*-infected macrophages treated with PEG-catalase 500 U/ml; MØ inf+E, *Leishmania*-infected macrophages treated with EGCG 12 µM; MØ inf+E+PEG-CAT, *Leishmania*-infected macrophages treated with EGCG (12 µM) and PEG-catalase (500 U/ml); G/GO, glucose+glucose oxidase. *Leishmania*-infected macrophages were either untreated (Panel C) or treated with PEG-catalase (Panel D), EGCG (Panel E) or EGCG+PEG-catalase (Panel F). The macrophages were fixed onto glass slides. The slides were stained with the Instant Prov hematological dye system and photographed (1000× magnification). * indicates a significant difference relative to *Leishmania*-infected macrophages (*p*<0.05); ** indicates a significant difference relative to *Leishmania*-infected macrophages (*p*<0.01); # indicates a significant difference relative to *Leishmania*-infected macrophages treated with 12 µM EGCG (*p*<0.05); ## indicates a significant difference relative to *Leishmania*-infected macrophages treated with 12 µM EGCG (*p*<0.01).

Previous studies suggest that EGCG induces H_2_O_2_ production, which may be linked to the cytotoxic effects of chemical treatments [22], [24], [35]. Thus, we tested H_2_O_2_ production in *L. braziliensis*-infected macrophages that were preincubated with polyethylene glycol (PEG)-catalase (500 U/ml). We determined that PEG-catalase protected *L. braziliensis* from EGCG-mediated inhibition (*p*<0.05) (Figure 8 panel A) and reduced ROS levels in *Leishmania*-infected macrophages treated with EGCG (*p*<0.05) (Figure 8B). EGCG treatment inhibited the intracellular amastigotes without any apparent cytotoxicity as evidenced by the intact cell morphology (Figure 8 C–F); the damage caused by increased ROS appeared to be selectively directed towards intracellular amastigotes.

### *In vivo* effects of EGCG in BALB/c mice infected with *Leishmania braziliensis*

**Figure 9:**
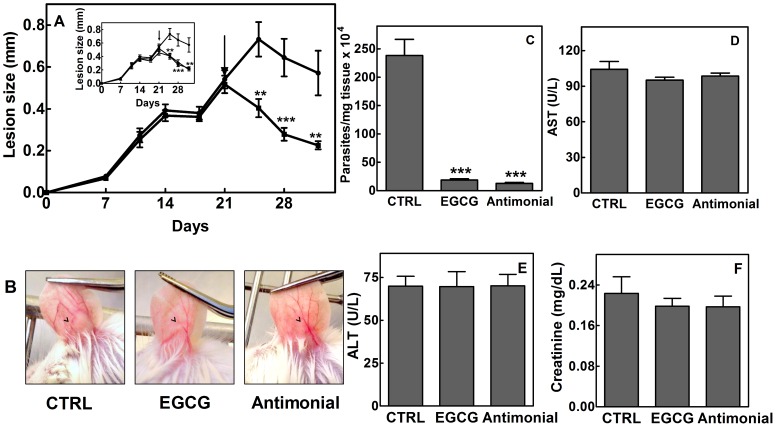
*In vivo* leishmanicidal effect of EGCG in *L. braziliensis*-infected BALB/c mice. The right ears of the mice were infected intradermally with 2×10^6^ *L. braziliensis* promastigotes. Panel A: Lesion development in the animals administered oral EGCG (100 mg/kg/day; closed square) or the control group orally administered sterile PBS (vehicle of EGCG; closed circle) once a day seven times a week. Arrow represents the initiation of treatment. Inset: Lesion development in animals that were administered oral EGCG (100 mg/kg/day; closed square) and the control groups, which were orally administered sterile PBS (vehicle; closed circle) or treated with intraperitoneal injections of meglumine antimoniate (30 mg/kg/day; open triangle) once a day seven times a week. The arrow represents the initiation of treatment. Panel B: Macroscopic evaluation of lesions (arrowhead) in untreated mice (left column), EGCG-treated mice (medium column), and meglumine antimoniate-treated mice (right column) at the end of the experiment (day 32). The arrowhead represents the lesion. Panel C: Parasite burden of *L. braziliensis*-infected BALB/c mice untreated or treated with EGCG (100 mg/kg/day) or meglumine antimoniate (30 mg/kg/day). Ear parasite loads were determined via a limiting dilution assay. Panels D–F: Toxicity parameters for the kidneys and liver. At the end of experiment, the mice were euthanized, and serum samples were collected for colorimetric determination of aspartate aminotransferase (AST) (panel D), alanine aminotransferase (ALT) (panel E), and creatinine (panel F) concentrations as parameters of liver and kidney toxicity. Data are expressed as the mean ± standard error, *n* = 5 ears. [*** indicates a significant differences relative to the control group (*p*<0.001)]. (CTRL, control; antimonial, meglumine antimoniate).

To assess the efficacy of EGCG *in vivo*, the ears of BALB/c mice were intradermally infected with 2×10^6^ *L. braziliensis* promastigotes, and the mice were treated orally with EGCG (100 mg/kg/day). As shown in Figure 9A and 9B, the oral administration of EGCG reduced the lesion size compared with the control group (*p*<0.001).

Interestingly, EGCG oral treatment significantly reduced the parasite burden (92.1% of reduction; *p*<0.001) compared with the control group (Figure 9C). However, no significant differences in lesion size (60.5% and 64.0%, respectively; Figure 9 panel A inset and panel B) and parasite load (92.1% and 94.7%, respectively; Figure 9 panel C) were observed between the infected mice treated with EGCG or meglumine antimoniate. Furthermore, no significant differences in serum ALT (Figure 9D), AST (Figure 9E) and creatinine (Figure 9F) levels were observed between mice treated with EGCG and untreated mice (the control group).

## Discussion

EGCG is the most abundant and widely studied flavonoid. EGCG has generated considerable interest as a pharmaceutical compound due to its wide range of therapeutic activities [16], [36], such as those exhibited against *T. cruzi* [18], [37]. In the present study, we demonstrated the effect of EGCG *in vitro* on *L. braziliensis* promastigotes and intracellular amastigote forms and *in vivo* on *L. braziliensis*-infected BALB/c mice. In addition, we describe the EGCG mechanism against *Leishmania braziliensis* promastigotes and intracellular amastigotes.

EGCG inhibited *L. braziliensis* promastigote viability in a dose-dependent manner, achieving 80.7% inhibition upon treatment with 500 µM EGCG. These results demonstrate the antileishmanial activity of EGCG against *L. braziliensis* promastigotes. Similar dose-dependent EGCG activities were observed in the promastigote and intracellular amastigote forms of *L. amazonensis* [19], [20]. The trypanocidal effects of EGCG against epimastigotes, amastigotes and trypomastigotes have been reported [18], [37].

The treatment of intracellular amastigotes with EGCG resulted in a time- and dose-dependent inhibitory effect, with IC_50_ values of 3.7 and 3.4 µM at 24 and 72 h, respectively, and a selectivity index of 103.3 and 149.5 at 24 and 72 h, respectively. The biological efficacy of a drug is not attributed to cytotoxicity when the selectivity index ≥10 [26], [38]. These results demonstrate the antileishmanial activity of EGCG against *L. braziliensis* amastigotes.

The antileishmanial potency of EGCG was greater than that of miltefosine, which has been successfully used for the treatment of New World leishmaniasis [39]–[42], with an IC_50_ value of 5.40 µM at 72 h for *L. braziliensis* and a selectivity index of 17.2 [42].

It has been demonstrated that the effectiveness of inhibitor compounds may depend on the developmental stage of the parasite. For instance, Santos et al. [43] demonstrated that *L. amazonensis* amastigotes developing within macrophages are more sensitive to HIV aspartyl peptidase inhibitors than promastigotes developing in culture medium, which may explain why promastigotes were less susceptible to EGCG than intracellular amastigotes.

Another possible explanation for the distinct action of EGCG on promastigotes alone and on amastigotes in an intracellular environment is the idea that macrophages could accumulate higher levels of EGCG. Accordingly, it was shown in *L. infantum* that lower concentrations of HIV-1 protease inhibitors are necessary to exert a pronounced effect against intracellular amastigotes compared to axenic amastigotes [44].

ROS are generated in cells to fight pathogenic infections. ROS are also generated in response to various drugs. This mechanism is the basis of various antiprotozoal medications used to combat parasites in infected cells. Importantly, the ability of a drug to generate ROS, which result in the destruction of cellular macromolecular components, can be modulated to derive maximal effects [45]. In this study, EGCG increased H_2_O_2_ generation in promastigotes in a dose-dependent manner, and H_2_O_2_ production directly correlated with the percent inhibition of viable promastigotes. Our results are consistent with results from Fonseca-Silva et al., who previously demonstrated that quercetin, the most common flavone in the human diet, induces ROS production in a dose-dependent manner in *L. amazonensis* [13].

In amastigotes from *Leishmania*-infected macrophages, EGCG increased ROS generation after 24 h, the shortest time resulting in infection index reduction (73% reduction), suggesting that increased ROS could be specific to intracellular amastigotes. The exposure of *L. amazonensis*-infected macrophages to diethyldithiocarbamate (DETC) [28] and quercetin [34] has been shown to increase superoxide anion and reactive oxygen species levels, respectively. These effects subsequently induce a severe reduction in the number of intracellular parasites and demonstrate the efficacy of ROS as an antimicrobial agent against intracellular parasites.

PEG-catalase significantly reduced EGCG-induced promastigote and intracellular amastigote death without apparent cytotoxicity to the EGCG-treated macrophages. Therefore, we postulate that EGCG-induced leishmanicidal activity occurs, at least in part, through ROS selectively directed towards promastigotes and intracellular amastigotes, thereby potentially altering the cellular redox status.

Mitochondria are essential cellular organelles that play a central role in energy metabolism. Mitochondria are critical for the survival of all cells. Maintenance of mitochondrial membrane potential (ΔΨ_m_) is vital for this metabolic process and cell survival [46], [47]. Studies have demonstrated that variations in ΔΨ_m_ induced by drugs are associated with cell survival in *T. cruzi* [12], [48], *Leishmania donovani* [47] and *L. amazonensis* [13], [20], [49]. We demonstrated altered ΔΨ_m_ in the EGCG-treated promastigotes. The collapse of ΔΨ_m_ results from ROS added directly *in vitro* or induced by chemical agents [50], [51]. Therefore, we suggest that EGCG exerts its antileishmanial effect on *L. braziliensis* promastigotes via H_2_O_2_ production followed by a loss of ΔΨ_m_.

Mitochondria are responsible for respiration and oxidative phosphorylation in eukaryotes, including trypanosomes. Mitochondria provide ATP through respiratory-coupled oxidative phosphorylation [52]. A decrease in ΔΨ_m_ suggests increased proton permeability across the inner mitochondrial membrane, thereby decreasing ATP synthesis and resulting in parasite death. We also demonstrated that EGCG reduced intracellular ATP concentrations, thereby promoting a global breakdown in the parasite metabolism.

The oxidative imbalance that leads to a decrease in ΔΨ_m_, thus reducing the intracellular ATP concentration, could occur through the reduction of trypanothione reductase (TR) activity. TR is an enzyme that participates in ROS detoxification of trypanosomatids and could be inhibited by EGCG. This trypanothione-dependent pathway is unique to the parasite and absent in the mammalian host [53], [54]. This effect has been demonstrated by the treatment of *T. cruzi* with eupomatenoid-5 [55]. Further studies should be conducted to demonstrate this inhibition.

To date, an ideal experimental model for *Leishmania braziliensis* infection is unavailable. BALB/c mice infected with *L. braziliensis* in the ear dermis serve as a model of localized cutaneous leishmaniasis. These mice develop nodular and ulcerated lesions that spontaneously heal within 10 weeks [27], [56].

The lack of affordable therapy necessitates the development of novel antileishmanial therapies. Here, we demonstrated that oral EGCG treatment reduces the lesion size and parasite load *in vivo*. In addition, EGCG did not alter serological toxicology markers, such as aminotransferases and creatinine, in the infected mice. However, further specific toxicity studies, such as genotoxicity, should be performed.

EGCG decreased the lesion size and parasite load without compromising the overall health of the infected mice. These results are encouraging and suggest that EGCG should be further studied as a potential leishmaniasis chemotherapy. Additionally, studies should be conducted to determine the ideal dose and therapeutic regimen.

In conclusion, our study suggests that EGCG displays leishmanicidal effects against the promastigote and amastigote forms of *L. braziliensis*. As part of the EGCG mechanism of action, ROS production decreases ΔΨ_m_ and reduces intracellular ATP concentrations, thereby promoting parasite death. Furthermore, our data suggest that EGCG is orally effective in the treatment of *L. braziliensis*-infected BALB/c mice without altering serological toxicology markers.
